# 

**Published:** 1892-05

**Authors:** 


### TABLE OF CONTENTS.

**ORIGINAL COMMUNICATIONS.** PAGE

The Dental Protective Association: What it has done for Dentists, and its Future Work in their Behalf. By LOUIS JACK, D.D.S. . . . . . . . . 321

Alveolar Abscess. By EDWARD C. KIRK, D.D.S. . . . . . . . . . . . . 332

Odontalgia. By CHARLES P. BRIGGS, M.D., D.M.D. . . . . . . . . . . 338

Dentistry One Hundred Years Ago. By WILLIAM DUNN, JR., D.D.S., Florence, Italy . . . . . . . . . . . . . . . . . . . . . . . . 344

A Gold Crown with Perfect Articulating Surface. By DR. L. C. BRYAN, Basel, Switzerland . . . . . . . . . . . . . . . . . . . . . . 347

History of Dentists who have graduated under Drs. Harwood and Tucker. By DR. E. G. TUCKER . . . . . . . . . . . . . . . . . . . . . 348

Replacing Porcelain Facings. By E. B. WHITE, D.D.S., Syracuse, N. Y. 349

**REPORTS OF SOCIETY MEETINGS.**

New York Odontological Society . . . . . . . . . . . . . . . . . . 351

American Dental Association . . . . . . . . . . . . . . . . . . . . 360

Odontological Society of Pennsylvania . . . . . . . . . . . . . . . 365

Harvard Odontological Society . . . . . . . . . . . . . . . . . . . 374

Central Dental Association of Northern New Jersey . . . . . . . . . . 380

**EDITORIAL.**

English Criticism . . . . . . . . . . . . . . . . . . . . . . . . 393

American Dental Association . . . . . . . . . . . . . . . . . . . . 396

**CURRENT NEWS.**

Dental Society of the State of New York . . . . . . . . . . . . . . 397

American Dental Association—Special Notice . . . . . . . . . . . . . 398

Union Meeting of the Pennsylvania and New Jersey State Dental Societies 398

Illinois State Dental Society . . . . . . . . . . . . . . . . . . . 398

Chicago Dental Society . . . . . . . . . . . . . . . . . . . . . . 399

Patents issued . . . . . . . . . . . . . . . . . . . . . . . . . . 399

American Medical Association . . . . . . . . . . . . . . . . . . . 399

**SELECTIONS.**

Abstracts from "The Journal of the British Dental Association," March, 1892 400

Alcodiform in capping Nerves . . . . . . . . . . . . . . . . . . . 404

